# Hot-Carrier Damage in N-Channel EDMOS Used in Single Photon Avalanche Diode Cell through Quasi-Static Modeling

**DOI:** 10.3390/mi15020205

**Published:** 2024-01-30

**Authors:** Alain Bravaix, Hugo Pitard, Xavier Federspiel, Florian Cacho

**Affiliations:** 1 IM2NP UMR 7334, REER-ISEN Méditerranée, Place G. Pompidou, 83000 Toulon, France; hugo.pitard@yncrea.fr; 2 ST Microelectronics, 850 rue Jean Monnet, BP16, 38926 Crolles, France; xavier.federspiel@st.com (X.F.); florian.cacho@st.com (F.C.)

**Keywords:** hot carriers, interface traps, electron trapping, slow traps, extended drain, MOSFETs source-drain resistance, mobility reduction, SPAD, smart power

## Abstract

A single photon avalanche diode (SPAD) cell using N-channel extended-drain metal oxide semiconductor (N-EDMOS) is tested for its hot-carrier damage (HCD) resistance. The stressing gate-voltage (V_GS_) dependence is compared to hot-hole (HH) injection, positive bias temperature (PBT) instability and off-mode (V_GS_ = 0). The goal was to check an accurate device lifetime extraction using accelerated DC to AC stressing by applying the quasi-static (QS) lifetime technique. N-EDMOS device is devoted to 3D bonding with CMOS imagers obtained by an optimized process with an effective gate-length L_eff_ = 0.25 µm and a SiO_2_ gate-oxide thickness T_ox_ = 5 nm. The operating frequency is 10 MHz at maximum supply voltage V_DDmax_ = 5.5 V. TCAD simulations are used to determine the real voltage and timing configurations for the device in a mixed structure of the SPAD cell. AC device lifetime is obtained using worst-case DC accelerating degradation, which is transferred by QS technique to the AC waveforms applied to N-EDMOS device. This allows us to accurately obtain the AC device lifetime as a function of the delay and load for a fixed pulse shape. It shows the predominance of the high energy hot-carriers involved in the first substrate current peak during transients.

## 1. Introduction

**Figure 1:**
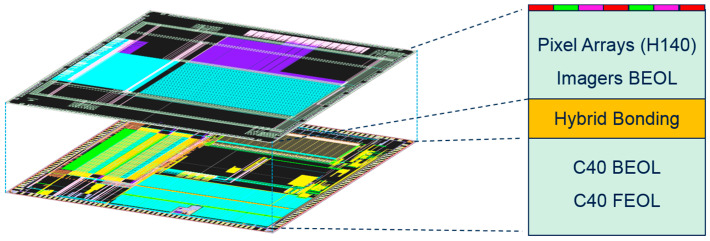
3D integration of pixel arrays and CMOS image sensors at the top tier H140 (L_nom_ = 140 nm) stacked by hybrid bonding with a bottom tier in C40 (L_nom_ = 40 nm) for the logic part of the image sensor with back end of the line (BEOL) and front end of the line (FEOL) parts.

Single photon avalanche diodes (SPAD) are prone to better integration with the down scaling of circuits for high sensitivity imagers [1], widely used in embedded submicronic systems [2]. With the limitations induced by the pitch reduction and the increase in the number of horizontal interconnections, the die architecture has moved to 3D stacking of heterogeneous systems [3]. The architecture is composed (Figure 1) of the vertical assembly of the pixel arrays, the image signal processor and the logic parts. The hybrid bonding through silicon via (TSV) allows the SPAD to gain in BEOL density with the stacking of different CMOS technologies. It can include a memory chip inserted as a third tier between the pixel array and logic chip, which enables high speed readout [4]. Stronger integration has been proposed with an ultra-thin body (UTB) and a fully depleted silicon on insulator (FDSOI) node [2], which shows a good sensitivity for sensing activity. In this case, a better trade-off between performance and consumption is obtained [3]. However, the need for reliability evaluation is required with the use of different processing options. This can be studied by focusing on the benefits of using thin/thick gate-oxide (GO1/GO2), a proper gate-length (L_G_) and supply voltage (V_DD_) [5]. A controlled lateral field (E_Lat_) is needed [6], alongside an adapted source/drain terminal [7]. Hot-carrier damage (HCD) [8], bias temperature instability (BTI) [9] and hard breakdown (HBD) [10] are the most limiting wear out mechanisms that reduce MOSFET lifetime [11] regarding AC voltage and environment impact [12].

For device lifetime determination in N-EDMOS, the use of DC stressing first facilitates the distinction between worst-case damage mechanisms [8,9,10]. However, this presents the need to obtain the real AC accelerating degradation rate with bias and timing corresponding to the voltage conditions applied to the device in its cell environment [11,12]. A better approach can be obtained by using the quasi-static (QS) technique once the AC voltage conditions have been checked by ELDO simulations.

After the introduction of the SPAD cell in Section 2, we give the description of the devices and experiments in Section 3. The aim of this work is to show that N-channel EDMOS reliability can be obtained by determining the high energy HCD that remains the dominant mechanism under On-mode (Section 4) with respect to BTI damage, HH injection and Off-mode damage. DC characteristics of substrate current I_SUB_ vs. V_GS_ exhibit a second hump due to 2nd impact ionization into the drift region. However, the DC-AC transfer using QS technique shows in Section 5 that the first I_SUB_ peak dominates the damage during AC transients. This is done according to an adaptive waveform shaping based on the ELDO simulations, which corresponds to the true signals applied to N-EDMOS device placed into the SPAD cell. Even if one considers hot-hole (HH) damage at low V_GS_ for QS lifetime extraction, it does not lead to a lifetime reduction in EDMOS device. This is due to the reduced E_Lat_ (V_DD_) condition in this EDMOS structure. It originates from the optimized N-EDMOS GO2 device (L_eff_ = 0.25 µm, T_ox_ = 5 nm) with maximum voltages V_DSmax_ at 10% of the nominal supply voltage V_DDnom_ = 5 V, while limiting V_GSmax_ = V_DSmax_/2.

## 2. The SPAD Cell with EDMOS Transistor

**Figure 2:**
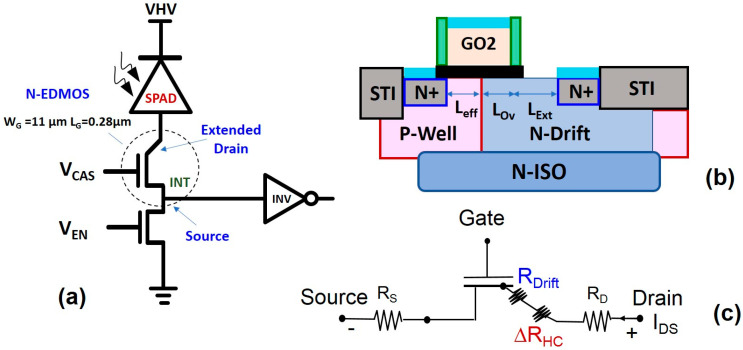
(**a**) Schematics of the SPAD cell with a N-channel EDMOS transistor inserted into the logic block. (**b**) Cut of the transistor structure composed of the N-Drift/p-well, N-ISO socket and lateral isolation by STI. (**c**) Illustration of the distinct resistance of the EDMOS transistor with R_S_, R_D_ from the source/drain terminal, the drift resistance R_Drift_ related to the overlap and extension length regions. HCD contribution from the gate-drain region leads to an additional resistance increase (ΔR_HC_) induced by interface traps and trapped charges.

The logic part of a SPAD cell has different processed devices; one is a drain-extended N-channel (N-EDMOS) transistor (Figure 2a), used for its switching capability. This results in a small on resistance (R_On_), a bias range with a sustainable large drain voltage (V_DS_), while the gate-voltage (V_GS_) is limited to V_DS_/2 to avoid breakdown of gate-oxide. This is related to the N-drift region (with respect to p-well) designed (Figure 2b) as a drain extension in series with channel, with an overlap length (L_ov_) with the gate and an extension (L_ext_) to the drain terminal. The optimization of the EDMOS architecture depends on several factors, such as the gate-oxide thickness (T_ox_), the lateral isolation by locally oxidized silicon (LOCOS) or by a shallow trench isolation (STI) [13,14]. It is also strongly related to the silicon [11] or silicon on insulator (SOI) substrate [15,16], the presence of a body buried layer [17] and the use of super junction [18]. These parameters lead to a different tradeoff between performance and device reliability. HCD can be used as it tests the N-EDMOS robustness due to the reduction in current drivability and device lifetime, induced by the source-drain resistance increase (R_SD_) (Figure 2c) in the drift region [14,15].

## 3. Devices and Experiments

The tested n-channel EDMOS devices originate from a third batch of advanced CMOS processing (Figure 2b) on a 300 mm wafer before the 3D bonding with the photodiodes. The transistor geometry is with nominal gate length (L_G_ = 0.5 µm) and gate-width (W_G_ = 11 µm), and gate oxide thickness (T_ox_ = 5 nm) (GO2) of SiO_2_. N-type (ISO) layer is used to isolate the channel from the substrate and the lateral isolation is conducted with shallow oxide trenches (STI). The effective channel-length is consequently L_eff_ = 0.25 µm = L_G_/2 while N-drift has an extended length defined by L_Drift_ = L_ov_ + L_ext_ where L_ov_ = 0.25 µm. Supply voltage is optimized for smart power application between V_DDnom_ = 5 V and V_DSmax_ = 5.5 V, where V_GS_ is limited to V_GS_ = V_DS_/2 into the voltage range. Standard I-V characterizations are used with A4156C analyzer controlled in temperature, where DC and AC stressing are followed by I-V characterizations as measurement-stress-measurement (MSM) sequences. When history and recovery effects are studied, we use fast switching experiments with (low leakage) E5250A switching boxes and an 8110A pulse generator using a single linear mode I_DS_-V_GS_ characteristics in order to maximize the possible effect of recovery. As this latter was observed in 40 nm CMOS (C40) with very thin SiON gate-oxide (T_ox_ = 1.7 nm) [19] and in high-K metal gate (HKMG) with HfO2-SiON with small equivalent oxide thickness EOT = 1.6-2.2 nm [20], we have verified that no significant recovery occurs in GO2 N-EDMOS. This indicates that the use of medium gate-oxide T_ox_ = 5 nm (GO2) is appropriate for reducing the impact of the vertical electric field in the supply voltage range. Lifetime extraction is carried out for 10% in the reduction of saturated drain current measured at V_DS_ = V_GS_ = V_DDnom_/2. This is intentionally chosen here as it represents the worst case of measurement bias under HCD in N-EDMOS. This lifetime criterion is also required because it remains a relevant bias point regarding the switching activity under AC operation, in direct relation to the transistor delay and frequency impact involved in the digital cell environment [6].

## 4. Worst Case DC Degradation in N-EDMOS

One specificity of extended drain transistors is that the region of the high electric field is moved to the drift region, out of the channel, where carriers generated from impact ionization (II) are further accelerated in the overlap and extension lengths. This may generate a second hot spot, susceptible to defects creation in this zone to the STI region in LDMOS [7,13], or mainly in the overlap zone of EDMOS [14]. The multiple hot spots found around the STI and near LOCOS show a net impact on the linear on-resistance (R_On_ = V_DS_/I_DS_) depending on the V_GS_ value with respect to V_DS_. This can be explained by the contribution of HCD under single particle (SP) at medium V_GS_ and under multiple particle (MP) degradation at larger V_GS_ [13], which may involve self-heating when V_GS_ ≥ V_DS_ [14,21]. Recent works have shown that the contribution of secondary holes generated by II leads to additional HCD effect in high voltage (HV) devices [22,23,24]. In this case, the SP (hot carriers) and MP (cold carriers) degradation mechanisms induce a cumulative effect showing that cold holes take a significant role in damaged high-voltage LDMOS [25]. Low power digital applications using thin gate-oxide devices have shown that HC and BTI damage follow an interplay that can be described by a full V_GS_, V_DS_ mapping [26], modelled by an extended nonradiative multiphonon (NMPeq.+II) framework. This physical modeling has demonstrated the importance of secondary carriers and history effects with alternating HC, BTI and recovery effects at high V_GS_ [26,27]. This was observed particularly in the p-channel MOSFET (Si bulk) structure when hot holes are involved at high |V_GS_| > |V_DS_| [26].

**Figure 3:**
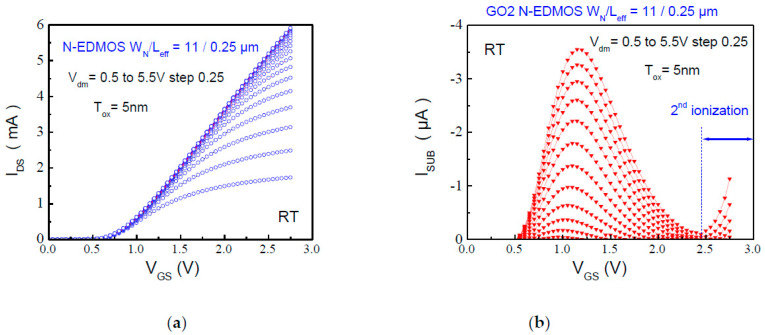
First step of the QS technique in L_eff_ = 0.25 µm N-EDMOS (**a**) Measurement of the I_DS_-V_GS_ curves done with a small V_DS_ step (V_DS_ = 0.5 V to 5.5 V step 0.25 V) in order to cover all the DC bias points submitted to a fresh device under (AC) operation. (**b**) Measurement of the substrate current I_SUB_—(V_GS_, V_DS_) for the same voltage range that shows the second hump at high V_GS_ due to 2nd impact ionization occurrence, that can reach avalanche mode depending on V_GS_ (V_DS_) magnitude.

As we want to deploy the QS technique to the special case of N-EDMOS placed into the SPAD cell, the first step is to characterize the substrate (I_SUB_) and drain current (I_DS_) dependence. It is conducted as a function of V_GS_, V_DS_ for all DC voltage conditions seen by the device in its AC environment. Figure 3a,b shows the I_DS_ and I_SUB_ currents representative of the II phenomenon in the transistor for a small incremental step of bias condition. We observe the channel current (Figure 3a) as the source of carriers able to trigger II at the drain, which is measured through the first hump of I_SUB_ (Figure 3b). These humps appear at much lower V_GS_ than in standard MOSFET and have a symmetrical source/drain structure. This intrinsic I_SUB_ curve is generally composed by the hot-hole population induced by the first (II) electron-hole pairs generation at the (first) peak lateral field entering the drain (N-Drift).

The departure of a second hump through the rise in I_SUB_ increasing V_GS_ (Figure 3b) indicates the existence of a second II hot spot located into the N-Drift, combined by the high I_DS_ value. This leads to an exponential rise in I_SUB_ in correlation to the Kirk effect [28]. The high electric field that peaks at the drift-drain junction induces I_SUB_ current rise, which has been recently modelled in a compact model for LDMOS transistor with similar structure [29]. We show in Figure 3b that I_SUB_ magnitude is much higher for the first I_SUB_ peak as a function of V_DS_ than its values obtained at V_GSmax_ increasing V_DS_. It indicates that the electric field is not as high as the one at low V_GS_ (1st I_SUB_ peak) in contrast to what was observed in LDMOS [29]; this is due to the limited voltage range in our N-EDMOS device.

**Figure 4:**
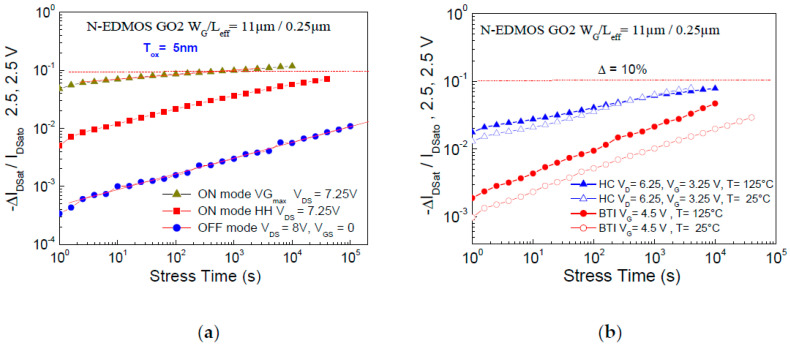
(**a**) Saturated drain current reduction at room temperature (RT) plotted as a function of V_GS_ stress between On mode at V_DS_ = 7.25 V under HH condition, V_GSmax_ = V_DS_/2 and Off mode at V_DS_ = 8 V and V_GS_ = 0. (**b**) Same criterion used to compare On mode damage at V_GSmax_ (V_DS_ = 6.25 V) and PBTI for V_GS_ = 4.5 V (V_DS_ = 0) between room temperature (RT = 25 °C) and 125 °C (DC) stressing.

In previous works [10,11,12], it has been shown that hot-hole (HH) damage at low V_GS_ stress and Off-mode damage at V_GS_ = 0 (high V_DS_) may trigger HBD in thin gate-oxide N-EDMOS localized in the gate-drain region above the N-Drift [10,11]. We have ensured that these behaviors are avoided in this present N-EDMOS device using thicker gate-oxide (5 nm) and longer gate-length [11]. HBD has been evidenced in this batch at V_DS_ = 16.5 V (V_GS_ = 0), which represents a much higher voltage condition than those used for On-mode damage (HCD and BTI) and Off-mode damage in this present work. It is shown in Figure 4a that V_GSmax_ condition is the worst-case for HCD in N-EDMOS in comparison to HH damage (V_Th_ ≤ V_GS_ ≤ 1.2 V) and Off-mode at V_GS_ = 0 (V_DS_ = 8 V) using long term DC stressing [30]. The comparison to positive BTI (PBTI) confirms, in Figure 4b, the lower degradation rate under BTI in N-channel EDMOS. Hence, choosing the classical V_GSmax_ = V_DSmax_/2 or a low V_GS_ for HH stressing condition [6,11] leads to a large difference in DC lifetime determination [30]. We show in the next section that the full QS technique applied to N-EDMOS is mainly based on the first I_SUB_ transients when one considers the real timing and voltage configurations submitted to the device placed into the SPAD cell.

## 5. Quasi-Static Lifetime Extraction

Accurate lifetime determination is known to be strongly dependent on the involved mechanisms in the device under operation [22,23,24,25,26] when one damage takes the lead, or several damages compete in parallel as a function of time. This is typically encountered between Off mode (V_GS_ = 0) and On-mode, whereas some damage may dominate for a short time and the others over the long term [8,9,10,11]. This depends on the progression of each mechanism (its nature, defect density and extension length) with time [8] and its possible effect on the accelerating degradation rate [9,10,11]. It may originate from interacting driven phenomena as the electric field [9,19] channel current [6,25] and thermal effects [26,27]. These effects can contribute to aggravation or relief in the device due to cumulating/compensating damage mechanisms that complicate the lifetime evaluation. This trend is generally observed in very thin gate-oxide as a history effect and recovery effect, which both depend on the gate and source/drain processing and device dimensions. It leads to specific behaviors that must be checked using AC stressing very close to the circuit functioning [30].

**Figure 5:**
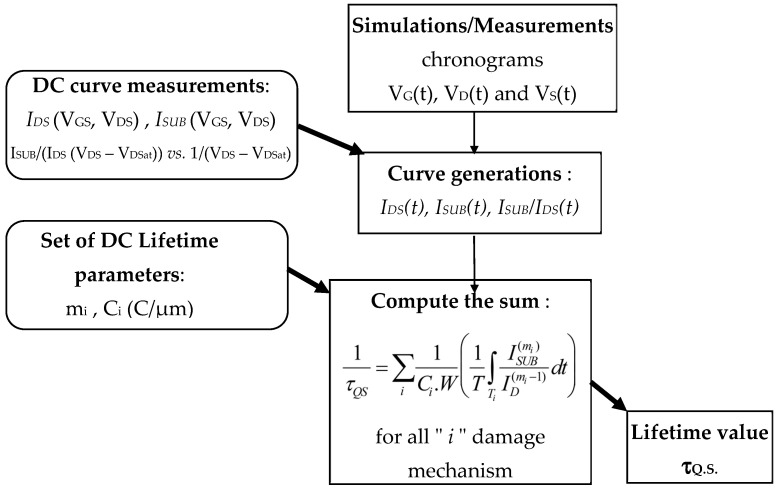
Program sequence of the QS lifetime extraction based on (1) V_D_, V_G_, V_S_ signal timing as a function of the N-EDMOS waveforms (2) the generation of the experimental curves from the DC I_DS_ (V_GS_, V_DS_) I_SUB_ (V_GS_, V_DS_) from Figure 3a,b which are transferred to every voltage condition found at each timing point of the chronogram. (3) Calculation of the QS lifetime with the set of lifetime parameters (m_i_, C_i_) as the sum of each damage mechanism “i”, of duration X_i_. This is moved automatically to the waveforms, i.e., as a function of V_DS_ = V_D_ − INT and V_GS_ = VCAS − INT (Figure 2a), for Off-mode (0 ≤ V_GS_ < V_Th_), HH stress (V_Th_ ≤ V_GS_ < 0.18 V_DS_), I_SUB_ stress (0.2 V_DS_ ≤ V_GS_ < 0.44 V_DS_) and V_GSmax_ stress (corresponding to 0.46 V_DS_ ≤ V_GS_ < 0.5 V_DS_). This enables in the final step to calculate the QS lifetime considering the duty cycle factor α_duty_ and pulse shape α_shape_ of the waveforms.

**Figure 6:**
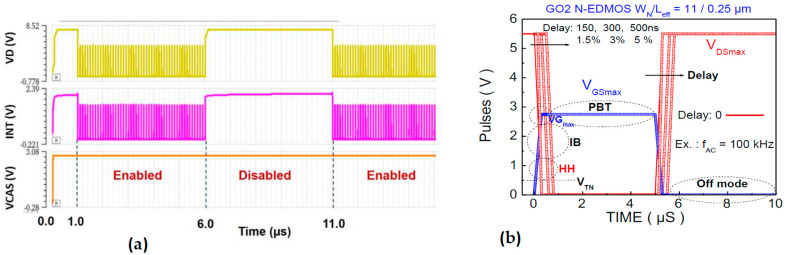
(**a**) ELDO potential simulation of timings for V_D_, V_S_ (INT) and V_G_ (VCAS) in N-EDMOS into the SPAD cell (**b**) Waveforms obtained from QS extraction as a function of delay = 0 to 5%, taking into account the corresponding V_GS_ (t) V_DS_ (t) variation allocated to each damage mechanism phase.

We have applied the QS technique [31,32,33], illustrated in Figure 5, by considering the timing results obtained by TCAD simulation (shown in Figure 6a) for the N-EDMOS device in the SPAD cell (Figure 2a). N-EDMOS device is switched in the cell with the potential variation of photodiode detector at its drain during enabled/disabled cycles [2], here with a frequency (f_AC_) of 100 kHz. Since the N-EDMOS source is switched by INT potential (Figure 2a), it varies V_DS_, V_GS_ applied to the transistor where V_GS_ = V_DS_/2 under the low SPAD illumination duration (Figure 6a), with a frequency of 10 MHz. This effect switches the transistor between On- and Off- mode through body effect on V_Th_ despite V_G_ (VCAS) being constant. Hence, the On-mode frequency (Figure 6a) chosen for TCAD is 10 MHz, while the longer Off mode is implied with 5 µs duration under low illumination. This enables us to obtain the low to high frequency range (Figure 6a,b), which would imply the possible influence of recovery effect (when V_DS_ = 0). This latter has not been observed using constant pulse shape experiments [30]. Therefore, we obtained AC results (Figure 6b) that conformed to the EDMOS waveforms independently of the frequency by fixing the pulse shape α_shape_ = t_r,f_/T_AC_ for a given delay. This enabled us to obtain the load variation range between the output and input of the pulsed transistor (i.e., between V_DS_ and V_GS_) into the SPAD cell.

The real waveform shape applied to N-EDMOS device into the SPAD cell has been simulated (Figure 6a) with the TCAD (ELDO) tool. This enables us to set up the parameters used for the V_GS_, V_DS_ pulse shaping and timing periods used by the QS methodology [32,33], as shown in Figure 5. The distinct damaging phases can be successively considered (Figure 6b) in order to assess the QS lifetime calculation. For each “i” damage mechanism effective in each duration phase, we compute τ_DC,i_ calculation under On-mode operation with:

(1) $$\frac{1}{\tau_{DC,i}}=\frac{1}{C_{i}.w}\frac{I_{SUB}^{\left(m_{i}\right)}}{I_{D}^{\left(m_{i}-1\right)}}$$

with:

- (m_HH_, C_HH_) corresponding to time duration under HH degradation where voltage satisfies V_Th_ ≤ V_GS_ < 0.18 V_DS_
- (m_IB_, C_IB_) corresponding to time duration under I_SUB_ peak degradation where we have 0.2 V_DS_ ≤ V_GS_ < 0.44 V_DS_
- (m_Vgmax_, C_Vgmax_) corresponding to time duration under V_GSmax_ degradation where we have 0.46 V_DS_ ≤ V_GS_ < 0.5 V_DS_

The calculation of the complete QS device lifetime is the sum of the integrated lifetime values obtained from each DC lifetime considered independently (with neither history effect nor recovery). Considering the dominant effect of high energy hot-carriers, the discretization of the (V_GS_, V_DS_) pulses is thus computed by the program with:

(2) $$\frac{1}{\tau_{QS}}=\sum_{i}\frac{1}{C_{i}.W}\left(\frac{1}{T}\int_{T_{i}}\frac{I_{SUB}^{\left(m_{i}\right)}}{I_{D}^{\left(m_{i}-1\right)}}dt\right)$$

**Figure 7:**
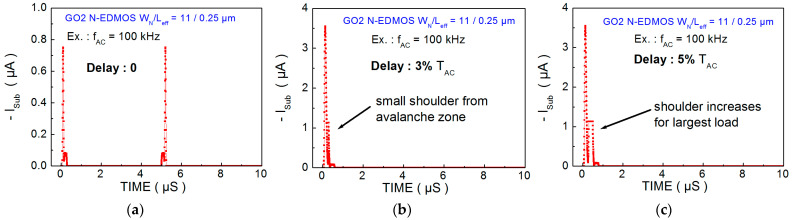
I_SUB_ (V_GS_, V_DS_, time) from a fresh N-EDMOS device transferred to the pulse waveforms as a function of the signal period (f_AC_ = 100 kHz) corresponding to the biased EDMOS placed into the SPAD cell (**a**) for no delay (**b**) for 3% delay and (**c**) for 5% delay.

Then, the program (Figure 5) builds up the I_DS_, I_SUB_ and (I_SUB_/I_DS_) time dependences, which one can loop for each delay value (Figure 6b). Results in Figure 7a show the symmetrical peaks of I_SUB_ (t) when no delay is involved (intrinsic case). For 3% delay value (typical digital application) and for 5% value at large load (Figure 7b,c), it induces I_SUB_ peaks only in the V_GS_ rising when V_DS_ is high. Since V_GS_ is limited to V_DS_/2, it makes the second I_SUB_ peak disappear. We note that a shoulder grows with the delay on the right side of the first I_SUB_ peak due to the increase in I_SUB_ (V_GS_) in the V_GSmax_ region, as previously shown in Figure 3b, in relation to E_Lat_ increase in the N-drift to drain contact region (Section 4).

**Figure 8:**
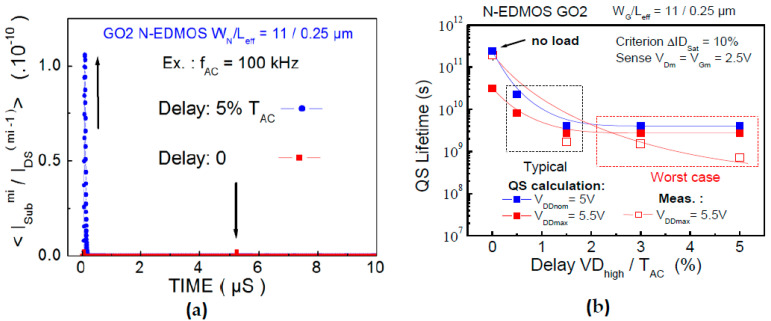
(**a**) Integrated (I_SUB_/I_DS_) ratio to the power of lifetime parameter (m_i_) with (1)–(2) for each damage region, which are plotted as a function of no delay and 5% delay. (**b**) Lifetime extraction for the full pulse waveform based on ΔI_DSat_ = 10% compared between QS calculation and the measurements in N-EDMOS into the SPAD cell, biased at V_DDnom_ and V_DDmax_.

Our previous results on lifetime extraction in the same technology [30] have used a simplification for the QS technique using the τ_DC_ calculation based on the power-law dependence with V_DS_. This was performed in order to compare the On-mode to Off-mode damage by its lifetime dependence expressed as τ_DC,i_ ∝ C_i_ V_DS_^−mi^. In this case, we have shown that the Off-mode and PBTI at V_GSmax_ stressing do not lead to a significant impact in the QS lifetime calculation [30], whereas the On-mode dominates the QS device lifetime. We have considered, here, the full QS modeling during On-mode (Figure 8a,b) based on the complete calculation with (1)–(2), i.e., for HH, I_SUB_ and V_GSmax_ stressing phases. The delay range is set between no load and large load (5% delay) to enhance HCD. QS lifetime plot obtained by the single (I_SUB_ ^mi^/I_DS_ ^(mi−1)^) peak at the V_GS_ rising front, and it contains no additional shoulder, as seen in I_SUB_ (Figure 7b,c). This indicates the lack of the weighting lifetime factors (m_i_, C_i_) effect in (2) for these timings in the V_GSmax_ voltage region. This suggests no significant incidence of HH sequence throughout the AC pulsing. This consequently leads to the QS lifetime calculation (Figure 8b), which is mainly dependent on (m_IB_, C_IB_) as a function of delay, whereas device lifetime is clearly reduced for 3–5% delay (worst case). In contrast, the QS result for no load is far less degrading, showing a limited scatter between the QS lifetime extraction and the measurements performed at the voltage condition V_DSmax_, V_GSmax_. These results confirm the validity of the QS technique for lifetime determination in the target [V_DDnom_,V_DDmax_] applied to the N-EDMOS placed into the SPAD cell.

With the help of the timing simulation by TCAD, there is a benefit to accurately matching the real configuration of voltage conditions of the N-EDMOS device that exceeds the 10-year lifetime under AC operation.

## 6. Conclusions

N-EDMOS device used in SPAD cell for future 3D stacking of CMOS imagers has been studied under AC stressing corresponding to its real AC operation configuration. The application of the QS technique shows that the device lifetime expressed as a function of each DC degradation mechanisms involved during V_GS_, V_DS_ pulsing leads to the predominance of the first I_SUB_ peak related to high energy hot-carriers. With the use of TCAD simulations for determining the real timing and voltage configurations, the QS technique shows that AC device lifetime is mainly dependent on the HCD that lies during V_GS_ rising transients. This indicates a much smaller contribution of cold carriers, HH damage and Off-mode damage during V_GS_ pulsing due to a limited voltage range V_GSmax_ = V_DSmax_/2. The main reason is the small effect of the weighting factors (m_i_, C_i_) in the full QS lifetime calculation, due to the real voltage and timing span involved during N-EDMOS device operation. It is thus confirmed that this processed N-EDMOS device (L_eff_ = 0.25 µm, T_ox_ = 5 nm, V_DDmax_ = 5.5 V) exceeds the 10-year AC lifetime for this mission profile. This guaranties that the designed N-EDMOS placed in SPAD cell is a good candidate for the future 3D stacking of CMOS imagers using hybrid bonding with the logic circuits.

## Data Availability

Data available on request.

## Abbreviations

BEOL	Back-End of the Line
BTI	Bias Temperature Instability
EDMOS	Extended-Drain Metal Oxide Semiconductor
HBD	Hard Breakdown
HCD	Hot-Carrier Damage
HH	Hot Hole
HKMG	High-K Metal Gate
II	Impact Ionization
LOCOS	Locally Oxidized Silicon
MOSFET	Metal-Oxide-Semiconductor Field Effect Transistor
PBTI	Positive Bias Temperature Instability
QS	Quasi-Static
STI	Shallow Trench Isolation
SPAD	Single Photon Avalanche Diode
FDSOI	Fully Depleted Silicon on Insulator
TSV	Through Silicon Via
